# Meeting 24-h movement guidelines and markers of adiposity in adults from eight Latin America countries: the ELANS study

**DOI:** 10.1038/s41598-022-15504-z

**Published:** 2022-07-05

**Authors:** Gerson Ferrari, Carlos Cristi-Montero, Clemens Drenowatz, Irina Kovalskys, Georgina Gómez, Attilio Rigotti, Lilia Yadira Cortés, Martha Yépez García, Maria Reyna Liria-Domínguez, Marianella Herrera-Cuenca, Miguel Peralta, Adilson Marques, Priscila Marconcin, Roberto Fernandes da Costa, Ana Carolina B. Leme, Claudio Farías-Valenzuela, Paloma Ferrero-Hernández, Mauro Fisberg

**Affiliations:** 1 Escuela de Ciencias de la Actividad Física, el Deporte y la Salud, Universidad de Santiago de Chile (USACH), Las Sophoras 175, Estación Central, Santiago, Chile; 2 Laboratorio de Rendimiento Humano, Grupo de Estudio en Educación, Actividad Física y Salud (GEEAFyS), Universidad Católica del Maule, Talca, Chile; 3 IRyS Group, Physical Education School, Pontificia Universidad Catolica de Valparaiso, Valparaiso, Chile; 4 Division of Sport, Physical Activity and Health, University of Education Upper Austria, Linz, Austria; 5 Carrera de Nutrición, Facultad de Ciencias Médicas, Pontificia Universidad Católica Argentina, Buenos Aires, Argentina; 6 Departamento de Bioquímica, Escuela de Medicina, Universidad de Costa Rica, San José, Costa Rica; 7 Centro de Nutrición Molecular y Enfermedades Crónicas, Departamento de Nutrición, Diabetes y Metabolismo, Escuela de Medicina, Pontificia Universidad Católica, Santiago, Chile; 8 Departamento de Nutrición y Bioquímica, Pontificia Universidad Javeriana, Bogotá, Colombia; 9 Colégio de Ciencias de la Salud, Universidad San Francisco de Quito, Quito, Ecuador; 10 Instituto de Investigación Nutricional, La Molina, Lima Peru; 11 Faculty of Health Sciences, Universidad Peruana de Ciencias Aplicadas (UPC), Lima, Peru; 12 Centro de Estudios del Desarrollo, Universidad Central de Venezuela (CENDES-UCV)/Fundación Bengoa, Caracas, Venezuela; 13 CIPER, Faculdade de Motricidade Humana, Universidade de Lisboa, Lisbon, Portugal; 14 ISAMB, Faculdade de Medicina, Universidade de Lisboa, Lisbon, Portugal; 15 Faculty of Human Kinetics, University of Lisbon, Lisbon, Portugal; 16 Physical Education Department, Health Sciences Centre, Universidade Federal do Rio Grande do Norte, Natal, Brazil; 17 Centro de Excelencia em Nutrição e Dificuldades Alimentaes (CENDA), Instituto Pensi, Fundação José Luiz Egydio Setubal, Hospital Infantil Sabará, São Paulo, Brazil; 18 Family Relations and Applied Nutrition, University of Guelph, Guelph, ON N1G 2W1 Canada; 19 Department of Nutrition, School of Public Health, University of São Paulo, São Paulo, Brazil; 20 Instituto del Deporte, Universidad de las Américas, 9170022 Santiago, Chile; 21 Facultad de Educación y Cultura, Universidad SEK, Santiago, Chile; 22 Departamento de Pediatria da, Universidade Federal de São Paulo, São Paulo, Brazil

**Keywords:** Diseases, Risk factors

## Abstract

This study aimed to compare compliance with 24-h movement guidelines across countries and examine the associations with markers of adiposity in adults from eight Latin American countries. The sample consisted of 2338 adults aged 18–65 years. Moderate-to-vigorous physical activity (MVPA) and sedentary behavior (SB) data were objectively measured using accelerometers. Sleep duration was self-reported using a daily log. Body mass index and waist circumference were assessed as markers of adiposity. Meeting the 24-h movement guidelines was defined as ≥ 150 min/week of MVPA; ≤ 8 h/day of SB; and between 7 and 9 h/day of sleep. The number of guidelines being met was 0.90 (95% CI 0.86, 0.93) with higher value in men than women. We found differences between countries. Meeting two and three movement guidelines was associated with overweight/obesity (OR: 0.75, 95% CI 0.58, 0.97 and OR: 0.69, 95% CI 0.51, 0.85, respectively) and high waist circumference (OR: 0.74, 95% CI 0.56, 0.97 and OR: 0.77, 95% CI 0.62, 0.96). Meeting MVPA and SB recommendations were related to reduced adiposity markers but only in men. Future research is needed to gain insights into the directionality of the associations between 24-h movement guidelines compliance and markers of adiposity but also the mechanisms underlying explaining differences between men and women.

## Introduction

Epidemiologic studies showed that sleep^1^, sedentary behavior^2^, and moderate-to-vigorous intensity physical activity (MVPA)^3^ are associated with reduced morbidity and mortality in adults independent of age and sex. Given that a 24-h period consists of three types of movement behaviors (i.e., physical activity, sedentary behavior, and sleep)^4^, monitoring the levels and patterns of these behaviors is essential for health surveillance and for developing future preventative health strategies. Also, identifying those at risk due to unhealthy movement behavior patterns give a valuable information to tail future interventions as well as to permit an equitable allocation and provision of economic resources^5^.

Growing evidence shows that high MVPA, low sedentary behavior, and adequate sleep duration are collectively associated with a range of health benefits, such as lower body mass index and low waist circumference^6,7^. Based on the emerging evidence and a better understanding of the importance of considering these behaviors holistically, new public health guidelines that combine recommendations for physical activity, sedentary behavior, and sleep have been issued in many countries^7–12^. In addition, the World Health Organization has recently shown similar guidelines, but without a sleep recommendation for adults^13,14^. Determining individuals meeting these new public health guidelines is necessary to notify future health promotion and illness prevention policies and interventions. However, most previous studies have been conducted in the middle- and high-income countries^15–17^.

In the last two decades, Latin America has undergone important health and epidemiological transitions experiencing fast socioeconomic growth and lifestyle transformation^18^. This situation has been associated with an increase in diverse health risk factors but mainly overweight and obesity^19^. Alarming rates of overweight (32.0%) and obesity (19.6%) have been described in the Latin American countries, and these rates are projected to increase to 38.1 and 43.6%, respectively, by 2030^20^. Furthermore, physical inactivity prevalence in Latin America is the highest reported worldwide^21^ and average sedentary behavior exceeds nine hours per day^22^.

A recent systematic review on 24-h movement guideline adherence and the relationship with markers of adiposity found five studies with adults from high-income countries (i.e., United States, Canada, Australia, Denmark, and Ireland)^6^. The authors showed that adults meeting all three guidelines (i.e., physical activity, sedentary behavior, sleep) had lower body mass index and waist circumference compared with those meeting none of the guidelines^6^. However, additional research is warranted to expand the knowledge on the associations of compliance with 24-h movement guidelines and body weight in adults from Latin America.

Multi-country data on compliance with 24-h movement behaviors is nonexistent in Latin America due mainly to the wide variety of sampling strategy used. Besides, the lack of standardized and validated measurements further makes it difficult to evaluate inter-country differences in compliance with 24-h movement behaviors in adults across Latin American countries. Thus, data collected in a single country or subnational region may show limited variance and results^23–25^. Examining data in different cultural and economic contexts can improve the understanding of the generalizability of the results. Therefore, the purposes of this study were (a) to compare compliance with 24-h movement guidelines across eight Latin American countries and (b) examine the associations between compliance with 24-h movement guidelines and markers of adiposity in adults. We hypothesized that (a) the number of guidelines being met and combinations of them would differ in Latin American adults across countries and (b) the number of guidelines being met and combinations of them would be inversely associated with markers of adiposity in adults from Latin America.

## Methods

### Study design and participants

The Latin American Study of Nutrition and Health (Estudio Latinoamericano de Nutrición y Salud; ELANS) was an observational, epidemiological, cross-sectional study conducted in eight Latin American countries, including Argentina, Brazil, Chile, Colombia, Costa Rica, Ecuador, Peru, and Venezuela that focused only on urban areas. The data used in the present study were derived from the 2014–2015 cycle of the ELANS (https://es.elansstudy.com/) international database. A detailed description of the study design and sampling methodology can be found elsewhere^26,27^. The overarching ELANS protocol was approved by the Western Institutional Review Board (#20140605) and is registered at ClinicalTrials.gov (#NCT02226627). The ethical committee from each local institutional review board approved the ELANS study. The participants provided written informed consent/assent before data collection. All aspects of the study were in accordance with the Declaration of Helsinki.

The study was conducted using a complex and multistage cluster-stratified sampling design, representing all regions for each country and randomly selecting the main cities according to probability proportional to size method^26,27^. The sampling size required for essential accuracy was considered with a 95% confidence level, a maximum error of 3.5%, and a survey design effect of 1.75. Socioeconomic status was balanced based on national indices used in each country. Households were selected through systematic randomization. Selection of the respondent within a household was established using 50% of the sample next birthday, 50% last birthday, controlling quotas for sex, age, and socioeconomic level. Details have been previously published^27,28^.

A total of 10,134 individuals (15.0–65.0 years) were invited to participate in the ELANS study; however, 9218 (47.8% men) agreed to participate. A subsample of 2737 participants aged 15–65 years used accelerometers, representing 29.6% of the ELANS population^29,30^. Due to logistical and financial reasons, efforts were made to confirm that 30% of the sample was assessed using accelerometers^30–32^.

We excluded adolescents aged 15–17 years from the manuscript because this study focused on the adult population. In addition, the 24-h movement guideline recommendations are different for adolescents and adults^7,14^. Furthermore, we also excluded participants aged 65 years based on the age range (i.e., 18–64 years) according to the Canadian guidelines to distinguish adults from older adults^7^. Therefore, the sample in the present manuscript consists of 2338 participants (53.4% women) aged 18–64 years with valid accelerometer data representing 23.6% of the total ELANS sample.

The protocol used in this manuscript included data collected during two home visits. The first visit included an assessment of markers of adiposity. Additionally, a subsample of the designated respondents received instructions regarding using accelerometers to measure MVPA and sedentary behavior; they were also provided with diaries (to complete for seven consecutive days). The second visit, which included administering the questionnaire and retrieving the accelerometers, and diaries, occurred eight days after the first visit for participants who were provided with the accelerometers.

### Measures

Average time spent sedentary and in MVPA (min/day) was assessed using the GT3X + Actigraph (Fort Walton Beach, FL, United States). Prior studies have shown the GT3X + Actigraph to have acceptable technical reliability for sedentary behavior and MVPA^33,34^.

Participants were instructed to wear the accelerometer using an elasticized belt at the right hip (mid-axillary) during waking hours for seven consecutive days and to remove the device only when sleeping or for water activities (e.g., showering). The minimum wear time considered acceptable to be included in the study was five valid days, including least one weekend day, with at least 10 h of data following the removal for sleep time^35^. After excluding the nocturnal sleep period time, periods with at least 60 min of consecutive zero accelerometer counts were classified as non-wear time^36^.

Data were collected at a sampling frequency of 30 Hz, and subsequently downloaded using ActiLife Software (V6.0; ActiGraph, Pensacola, FL) in epochs of 60 s^37^. Sedentary behavior was defined as all activity below 100 counts per minute not including the sleep period and non-wear time. MVPA was defined as all behaviors greater or equal to 1952 counts per minute^38,39^. Sedentary behavior and MVPA were calculated and reported as minutes per day in the present analyses. Participants were instructed to complete a daily log, and to report the time they put the accelerometer belt on and the time when it was removed. Sleep duration was calculated by identifying non-wear time during valid accelerometer days, identifying the time between going to bed (removing the device) and waking up (wearing the device)^40^. Total sleep duration was presented as hours per night for analysis. Details on accelerometer data have been published elsewhere^29,30^.

In each country, the markers of adiposity (body weight, height, and waist circumferences) were measured while the participant wore light clothing and without shoes using standard procedures and equipment. Height was measured to the nearest 0.5 cm with the participant’s head in the Frankfort Plane. Body weight was measured to the nearest 0.1 kg after all outer clothing, heavy pocket items, shoes, and socks were removed with a calibrated electronic scale (Seca 213®, Seca Corporation Hamburg, Germany) using standard procedures^41^. Body mass index was calculated as body weight in kilograms divided by height in meters squared (kg/m^2^) and was categorized as underweight (< 18.5 kg/m^2^), normal weight (18.5–24.9 kg/m^2^), overweight (25.0–29.9 kg/m^2^) and obesity (≥ 30.0 kg/m^2^)^42^.

Waist circumference was measured with an inelastic tape to the nearest 0.1 cm. Measurements were taken midway between the lowest rib and the iliac crest on the horizontal plane, with the subject standing. We used the “substantially increased risk of metabolic complications (central obesity)” cutoff defined by World Health Organization, > 102 cm for men and > 88 cm for women^42^ in order to determine high waist circumference. These measurements of the markers of adiposity were completed in duplicate and the mean was considered for the analysis. Details on markers of adiposity have been published elsewhere^31,32^.

Sociodemographic variables were assessed using standard questionnaires during face-to-face interviews. Participants self-reported their age and were categorized into three age groups (18–34, 35–49, and 50–64 years) to obtain appropriate sample sizes. In addition, participants reported, sex (women, men), and marital status (single [not married, widowed, and divorced] and married). Further, education level data was divided into three strata: low (basic or lower), middle (elementary), and high (university degree) for all countries. Details on sociodemographic variables have been published previously^26,29^.

### Data analysis

Weighting was done according to sociodemographic characteristics, sex, socioeconomic level, and country^22,27^. Mean, 95% confidence interval (95% CI), and percentages were computed, as appropriate, to describe the variables by sociodemographic variables.

Each participant was categorized as either “meeting” or “not meeting” the time-specific recommendations outlined within the Canadian 24-h Movement Guidelines for adults aged 18–64 years. The recommendations were as follows: (1) engage in at least 150 min/week of MVPA; (2) spend ≤ 8 h/day in sedentary behavior; and (3) obtain between 7 and 9 h/day of sleep. The participants who met all three recommendations for MVPA, sedentary behavior, and sleep duration were categorized as meeting the integrated Canadian 24-h movement guidelines. The proportion of participants meeting the physical activity, sedentary behavior, and sleep duration recommendations by sex and by country were also calculated.

To obtain a complete profile of the 24-h compliance with movement guidelines, two variables were used in this study: (a) the a number of the guidelines being met (from 0 = “no guideline met” to 3 = “all three guidelines met”), and (b) combinations of the guidelines being met as a category variable (“none”, “MVPA guideline met”, “only sedentary behavior guideline met”, “only sleep guideline met”, “both the MVPA and sedentary behavior guidelines met”, “both the MVPA and sleep guidelines met”, “both the sedentary behavior and sleep guidelines met”, and “all three guidelines met”). Country differences in compliance with 24-h movement guidelines were tested using one-way ANOVA (dependent variable was the number of the guidelines being met) and Chi-square test (dependent variable was combinations of the guidelines being met).

The adjusted odds ratio (OR) and respective 95% CI were obtained from multivariable logistic regression models investigating the odds of two different outcomes: (a) overweight/obesity classification based on body mass index and (b) as above thresholds based on waist circumference^42^. The models are presented by sex adjusted for age, education level, marital status, and country. A significance level of *p* < 0.05 was used to interpret inferential analyses. All analyses were performed using SPSS V22 software (SPSS Inc., IBM Corp., Armonk, New York, NY, USA).

### Ethics approval and consent to participate

Ethical approval was provided by the Western Institutional Review Board (#20140605), and by the ethical review boards of the participating institutions. ELANS is registered at Clinical Trials #NCT02226627. Written informed consent/assent was obtained from all individuals, before commencement of the study.

## Results

Table 1 presents descriptive characteristics. The sample consisted of 2338 participants (Mean: 38.2, 95% CI 37.7, 38.7) with 53.4% women (95% CI 51.4, 55.4); 44.7% (95% CI 42.7, 46.7) of participants were aged < 35 years; over half (56.8%, 95% CI 54.7, 58.8) were classified as having a low education level; 52.5% (95% CI 50.5, 54.5) were married. The country with the lowest proportion of participants was Ecuador (10.7%, 95% CI 9.4, 11.9), and the highest was Brazil (19.5%, 95% CI 17.9, 21.1).

**Table 1:** Characteristics of participants (mean or percentage [95% CI]) according sociodemographic variables.

Characteristics	Mean or % (95% CI) N = 2338
**Sex**	
Women	53.4 (51.4, 55.4)
Men	46.6 (44.6, 48.6)
Age (years)	38.2 (37.7, 38.7)
**Age interval**	
18–34 years	44.7 (42.7, 46.7)
35–49 years	32.8 (30.9, 34.7)
50–64 years	22.5 (20.8, 24.2)
**Educational level**	
Low	56.8 (54.7, 58.8)
Middle	32.2 (30.3, 34.1)
High	11.0 (9.8, 12.3)
**Marital status**	
Single	47.5 (45.3, 49.7)
Married	52.5 (50.5, 54.5)
**Country**	
Argentina	11.2 (9.9, 12.5)
Brazil	19.5 (17.9, 21.1)
Chile	10.6 (9.3, 11.8)
Colombia	12.4 (11.1, 13.8)
Costa Rica	9.4 (8.2, 10.5)
Ecuador	10.7 (9.4, 11.9)
Peru	12.6 (11.2, 13.9)
Venezuela	13.8. (12.4, 15.2)

95% CI, 95% confidence interval.

The means (95% CI) of MVPA (min/day), sedentary behavior (min/day), and sleep duration (h/day) across the entire sample were 34.5 (95% CI 33.6, 35.5), 568.9 (95% CI 564.1, 573.3), and 9.6 (95% CI 9.4, 9.7), respectively. Mean weight and height was 72.6 kg (95% CI 72.0, 73.2), 162.8 cm (95% CI 162.1, 163.4), respectively. Mean adiposity markers were 27.3 kg/m^2^ (95% CI 26.7, 28.0), and 89.7 cm (95% CI 89.1, 90.3) for body mass index, and waist circumference (Table 2).

**Table 2:** Descriptive characteristic (mean and 95% CI) of participants by sex and country.

Variable	Total	Women	Men	Argentina	Brazil	Chile	Colombia	Costa Rica	Ecuador	Peru	Venezuela
MVPA (min/day)	34.5 (33.6, 35.5)	28.7 (27.6, 29.8)	41.0 (39.2, 42.9)	33.0 (20.2, 35.8)	32.8 (30.8, 34.9)	39.7 (36.8, 42.5)	34.2 (31.5, 36.8)	32.7 (29.1, 36.3)	39.3 (35.5, 43.1)	36.2 (33.2, 39.1)	30.6 (28.3, 32.9)
SB (min/day)	568.9 (564.1, 573.3)	558.9 (552.9, 564.8)	580.4 (572.9, 587.9)	581.3 (566.3, 596.3)	556.8 (545.9, 567.7)	550.1 (535.7, 564.5)	566.9 (554.7, 579.1)	563.1 (547.0, 579.3)	573.8 (559.1, 588.6)	593.4 (580.1, 606.7)	573.5 (561.1, 585.9)
Sleep duration (h/day)	9.6 (9.4, 9.7)	9.7 (9.5, 9.9)	9.4 (9.2, 9.6)	9.2 (8.7, 9.6)	9.3 (8.9, 9.6)	9.2 (8.8, 9.5)	10.2 (9.8, 10.6)	9.8 (9.3, 10.2)	9.6 (9.3, 9.9)	9.3 (9.0, 9.7)	10.1 (9.8, 10.4)
Body weight (kg)	72.6 (72.0, 73.2)	68.4 (67.6, 69.2)	77.43 (76.4, 78.3)	74.3 (72.1, 76.5)	74.86 (73.4, 76.3)	75.49 (73.5, 77.4)	68.1 (66.6, 69.6)	74.0 (71.9, 76.0)	68.9 (67.1, 70.7)	69.5 (67.8, 71.1)	74.6 (72.8, 76.4)
Height (cm)	162.8 (162.1, 163.4)	156.71 (156.3, 157.0)	169.7 (169.3, 170.2)	163.4 (162.2, 164.6)	164.9 (163.9, 165.7)	163.1 (161.9, 164.2)	162.8 (161.7, 163.9)	162.1 (160.8, 163.4)	159.2 (158.0, 160.4)	159.4 (158.4, 160.4)	165.4 (164.4, 166.5)
BMI (kg/m^2^)	27.3 (26.7, 28.0)	27.8 (27.5, 28.1)	26.8 (26.5, 27.14)	27.7 (27.0, 28.4)	27.5 (27.0, 28.0)	28.3 (27.6, 29.0)	25.7 (25.1, 26.2)	28.2 (27.4, 28.9)	27.2 (26.5, 27.9)	27.2 (26.7, 27.8)	27.2 (26.6, 27.8)
WC (cm)	89.7 (89.1, 90.3)	88.4 (87.6, 89.1)	91.2 (90.4, 92.0)	90.6 (88.7, 92.5)	88.9 (87.6, 90.1)	93.5 (91.9, 95.1)	85.7 (84.3, 87.0)	94.1 (91.9, 96.2)	88.3 (86.9, 89.7)	89.3 (87.9, 90.8)	89.4 (87.8, 90.9)

95% CI, 95% confidence interval; MVPA, moderate-to-vigorous physical activity; SB, sedentary behavior; BMI, body mass index; WC, waist circumference.

The number of participants meeting the 24-h movement guidelines was markedly higher in men than in women (1.02 [95% CI 0.98, 1.07] and 0.78 [95% CI 0.73, 0.82], *p* < 0.001). On average, the number of the guidelines being met in participants ranged from 0.74 (95% CI 0.65, 0.82, Venezuela) to 1.14 (95% CI 1.03, 1.24, Chile) with significant differences across countries (*p* < 0.001). Country differences (*p* < 0.001) were also found for the combinations of the guidelines being met. Specifically, around 0.3% of adults in Colombia met all three guidelines, while 4.6% of participants from Chile met all three guidelines. No sex differences were observed for compliance with all three movement guidelines (*p* = 0.126) (Table 3).

**Table 3:** Proportion of participants meeting the 24-h movement behavior recommendation.

Variables	Number of guidelines being met, mean (95% CI)	Combination of the guidelines being met (% [95% CI])						
		All three (MVPA, SB, and sleep duration) % (95% CI)	Only MVPA (min/week) % (95% CI)	Only sedentary behavior (h/day) % (95% CI)	Only sleep duration (h/day) % (95% CI)	MVPA + SB % (95% CI)	MVPA + sleep % (95% CI)	Sleep + SB % (95% CI)
Total	0.90 (0.86, 0.93)	1.6 (1.1, 2.1)	48.3 (46.2, 50.4)	22.0 (20.3, 23.8)	19.4 (17.8, 21.1)	12.6 (11.3, 14.1)	11.0 (9.7, 12.3)	2.0 (1.5, 2.6)
Men	1.02 (0.98, 1.07)	2.1 (1.3, 3.1)	58.7 (55.6, 61.7)	21.2 (18.7, 23.7)	22.8 (20.3, 25.3)	14.1 (12.1, 16.4)	14.8 (12.7, 17.0)	2.1 (1.4, 3.2)
Women	0.78 (0.73, 0.82)	1.2 (0.7, 2.0)	39.4 (36.6, 42.2)	22.7 (20.4, 25.1)	16.5 (14.4, 18.5)	11.3 (9.6, 13.2)	7.7 (6.3, 9.3)	1.9 (1.2, 2.8)
Sex differences	t = 7.25, *p* < 0.001	χ^2^ = (1,2180) = 2,517, *p* = 0.126						
Argentina	0.89 (0.79, 0.98)	0.8 (0.2, 3.0)	46.0 (39.7, 52.3)	17.2 (12.4, 21.9)	24.9 (19.6, 30.2)	7.9 (5.1, 12.0)	13.1 (9.4, 18.0)	0.8 (0.2, 3.0)
Brazil	0.86 (0.78, 0.93)	0.7 (0.2, 2.0)	45.1 (40.4, 49.8)	26.5 (22.3, 30.6)	15.9 (12.5, 19.3)	14.7 (11.7, 18.3)	7.6 (5.4, 10.5)	2.0 (1.1, 3.9)
Chile	1.14 (1.03, 1.24)	4.6 (2.5, 7.9)	58.8 (52.6, 64.9)	26.2 (20.7, 31.7)	27.9 (22.2, 33.5)	18.7 (14.3, 24.1)	20.1 (15.6, 25.6)	4.5 (2.5, 7.9)
Colombia	0.87 (0.78, 0.95)	0.3 (0.06, 1.9)	52.8 (47.0, 58.6)	20.4 (15.7, 25.0)	14.8 (10.7, 18.9)	11.5 (8.3, 15.7)	8.0 (5.4, 11.7)	0.4 (0.06, 1.9)
Costa Rica	0.89 (0.79, 0.98)	0.5 (0.08, 2.6)	44.0 (37.4, 50.6)	25.2 (19.5, 31.0)	20.3 (14.9, 25.6)	13.0 (9.2, 18.1)	9.0 (5.9, 13.6)	0.9 (0.02, 3.2)
Ecuador	0.95 (0.84, 1.05)	4.0 (2.1, 7.4)	51.8 (45.2, 58.3)	23.2 (17.7, 28.7)	19.4 (14.4, 24.3)	14.8 (10.8, 20.0)	14.0 (10.1, 19.1)	3.9 (2.0, 7.3)
Peru	0.90 (0.80, 0.99)	2.2 (0.1, 4.8)	49.3 (43.4, 55.2)	15.4 (11.1, 19.7)	24.6 (19.6, 29.5)	12.2 (8.6, 17.1)	12.9 (9.4, 17.4)	2.2 (1.0, 4.7)
Venezuela	0.74 (0.65, 0.82)	0.7 (0.2, 2.4)	41.5 (36.0, 47.1)	20.6 (16.0, 25.2)	12.5 (8.9, 16.2)	9.5 (6.7, 13.3)	6.9 (4.5, 10.3)	1.3 (0.5, 3.3)
Country differences	F = (7,2214) = 5.4, *p* < 0.001	χ^2^ = (7,2215) = 31,835, *p* = < 0.001						

MVPA =  ≥ 150 min/week; SB =  > 8 h/day; Sleep duration: between 7 and 9 h.

95% CI, 95% confidence interval; MVPA, moderate-to-vigorous physical activity; SB, sedentary behavior.

Compliance with two and three guidelines was negatively associated with overweight/obesity (OR: 0.75, 95% CI 0.58, 0.97 and OR: 0.69, 95% CI 0.51, 0.85) and having high waist circumference (OR: 0.74, 95% CI 0.56, 0.97 and OR: 0.77, 95% CI 0.62, 0.96) in adults after adjustment for age, education level, marital status, and country. These results, however, only remained in men. At the same time, there were no significant associations between meeting movement guidelines and adiposity measures in women. The associations were not significant between meeting sleep duration with markers of adiposity (Table 4).

**Table 4:** Adjusted odds ratios (OR [95% CI]) for the relationship between the 24-h movement behavior recommendation met and markers of adiposity by sex.

Variables	Total sample		Men		Women	
	Overweight/obesity OR (95% CI)	High waist circumference OR (95% CI)	Overweight/obesity OR (95% CI)	High waist circumference OR (95% CI)	Overweight/obesity OR (95% CI)	High waist circumference OR (95% CI)
**Number of guidelines met**						
0	Ref	Ref	Ref	Ref	Ref	Ref
1	0.85 (0.39, 1.84)	0.81 (0.35, 1.84)	0.59 (0.23, 1.52)	0.46 (0.12, 1.65)	1.80 (0.37, 8.56)	1.17 (0.35, 3.85)
2	0.75 (0.58, 0.97)*	0.74 (0.56, 0.97)*	0.64 (0.44, 0.92)	0.42 (0.26, 0.67)*	0.92 (0.64, 1.33)	1.02 (0.72, 1.44)
3	0.69 (0.51, 0.85)*	0.77 (0.62, 0.96)*	0.69 (0.50, 0.95)*	0.56 (0.38, 0.82)*	0.88 (0.51, 1.21)	0.90 (0.69, 1.17)
**Only MVPA (min/week)**						
Not guidelines met	Ref	Ref	Ref	Ref	Ref	Ref
Guidelines met	0.72 (0.60, 0.87)*	0.69 (0.57, 0.84)*	0.70 (0.53, 0.91)*	0.57 (0.36, 0.70)*	0.80 (0.58, 1.02)	0.84 (0.65, 1.07)
**Only SB (h/day)**						
Not guidelines met	Ref	Ref	Ref	Ref	Ref	Ref
Guidelines met	0.68 (0.48, 0.88)*	0.81 (0.70, 0.92)*	0.79 (0.72, 0.86)*	0.73 (0.55, 0.95)*	1.40 (1.02, 1.91)	1.50 (0.90, 2.10)
**Only sleep duration (h/day)**						
Not guidelines met	Ref	Ref	Ref	Ref	Ref	Ref
Guidelines met	0.81 (0.65, 1.00)	0.81 (0.63, 1.04)	0.81 (0.60, 1.09)	0.84 (0.56, 1.24)	0.82 (0.59, 1.14)	0.80 (0.58, 1.10)
**MVPA + SB**						
Not guidelines met	Ref	Ref	Ref	Ref	Ref	Ref
Guidelines met	0.55 (0.46, 0.64)*	0.70 (0.57, 0.83)*	0.66 (0.52, 0.80)*	0.54 (0.32, 0.92)*	1.39 (0.91, 2.13)	1.20 (0.82, 1.75)
**MVPA + sleep time**						
Not guidelines met	Ref	Ref	Ref	Ref	Ref	Ref
Guidelines met	0.88 (0.66, 1.17)	0.80 (0.58, 1.12)	0.89 (0.62, 1.28)	1.34 (0.81, 2.21)	0.88 (0.55, 1.40)	1.14 (0.72, 1.79)
**SB + sleep**						
Not guidelines met	Ref	Ref	Ref	Ref	Ref	Ref
Guidelines met	1.31 (0.61, 2.63)	1.19 (0.59, 2.38)	0.86 (0.35, 2.10)	0.68 (0.19, 2.37)	2.57 (0.73, 9.05)	1.71 (0.65, 4.46)

The models were adjusted for age, education level, marital status, and country.

OR, odds ratio; 95% CI, 95% confidence interval; MVPA, moderate-to-vigorous physical activity; SB, sedentary behavior.

## Discussion

The present study examined compliance with 24-h movement guidelines and the associations between compliance with 24-h movement guidelines and markers of adiposity in adults from eight Latin American countries. Overall, the proportion of adults complying with all 24-h movement behavior guidelines was small (1.6%). Besides, we found significant differences in compliance with the 24-h movement guidelines among countries. Furthermore, the number of guidelines being met was negatively associated with measures of adiposity in men but not in women.

This study is the first to examine compliance with 24-h movement guidelines in adults from Latin American countries. Thus, contributing to the previous literature by examining how meeting 24-h movement guidelines is associated with markers of adiposity. Two recent systematic reviews with compositional data analysis studies described that the composition of movement behaviors across the 24-h day (including physical activity, sedentary behavior, and sleep) was associated with all-cause mortality, markers of adiposity (i.e., body mass index, waist circumference, waist-to-hip ratio, and body fat), cardiometabolic biomarkers, and mental health^6,43^.

A negative association between the number of the guidelines being met and markers of adiposity was detected in Latin American adults. Our findings corroborate previous studies from high-income countries (for instance, the United States, Canada, and Denmark), which showed an increased likelihood for obesity in participants not meeting any of the movement guidelines^7,44,45^. A recent systematic review^7^ indicates that only two international studies^44,45^ examined the relationship between 24-h movement guidelines and markers of adiposity in adults. Both of which found that the distribution of time spent in 24-h movement behaviors was significantly associated with body mass index and waist circumference. For instance, in one study^45^ the strongest favorable association was found for the proportion of time spent in MVPA whereas unfavorable associations were found for the proportion of time spent in light physical activity and sedentary behavior. Our findings suggest that men from Latin America meeting more movement guidelines of MVPA, sedentary behavior, and adequate sleep were more likely to have lower body mass index and waist circumference. A relevant finding is that Latin American adults meeting more guidelines had healthier lifestyles such as sufficient MVPA, reduced sedentary activities, and adequate sleep. These healthy lifestyle behaviors may reduce the risk of overweight and obesity in adults. It should be acknowledged that current evidence for the association between the number of 24-h movement guidelines and adiposity in adults is still understudied in Latin America. Therefore, future research is recommended, including longitudinal study designs, to examine the potential direction of the associations.

We found that adults meeting both the physical activity and sedentary behavior guidelines were more likely to have lower odds ratios for body mass index and waist circumference than those meeting none. This finding suggests that a combination of sufficient MVPA and less sedentary behavior provides beneficial effects on maintaining healthy body weight in adults. Furthermore, this study did not detect a significant association between meeting MVPA plus sedentary behavior and sedentary behavior plus sleep with markers of adiposity, with the reference category of meeting none of the guideline in women. These findings are essential for understanding 24-h movement behaviors in Latin American adults and for establishing evidence-based intervention for preventing overweight and obesity.

The current findings suggest differences in the association between guideline adherence and body composition by sex. In international studies, low adherence to the MVPA and sleep duration recommendation has been previously described among women^46,47^. Particularly Latin American women seem to lag behind in MVPA compared with men in international research^24,29,48^. Increasing MVPA levels and sleep duration would be favorable for the population^49,50^ and would also increase the proportion of Latin American adults meeting the integrated 24-h movement recommendations. Intervention and public health promotion efforts to inspire MVPA and adequate sleep time, which enhance compliance with the 24-h movement guideline recommendations are essential in this group.

Several countries' development and release of evidence on 24-h movement guidelines^7–9^ characterize a new public health approach by providing specific recommendations for a healthy 24-h period, including time spent in physical activity, sedentary behavior, and sleep. The guidelines recognize that focusing on a single behavior has limitations and suggest that a combination of movement behaviors (physical activity, sedentary behavior, and sleep) matters for health and healthy development. Given that most Latin American adults do not met all movement guidelines, consideration should be given to presenting adequate behavior to multiple behavior change. Certainly, it has been theorized that a sequential rather than simultaneous approach to multiple movement behavior change may be more successful. Our findings illustrate important considerations that must be made about the content of Guidelines released in Latin America.

This study has several significant limitations and strengths. One limitation of the study is the cross-sectional design which cannot reveal the causality. Another potential limitation was that sleep duration was derived from the non-wear time of valid days. Sleep time was calculated using the device removal time during night time. This may overestimate sleep time, as participants may remove the device before effectively going to sleep and start wearing it sometime after waking up. However, to minimize this issue, participants were specifically instructed to only remove the device when going to sleep and wearing it immediately after waking up. Participants took notes in their daily logs, which were matched to identify potential problems. Wearing an accelerometer for too few hours per day can result in an underestimation of time spent in different physical activity intensity categories. Assessing physical activity for more hours per day can provide a more accurate picture of the true levels of physical activity and avoid misclassification bias^51,52^. Misclassification of physical activity can bias results of studies toward the null, alter the interpretations for dose–response relationships between physical activity and inactivity exposures and health outcomes, modify the proportion of adults meeting public health physical activity recommendations, and change the interpretations of intervention studies. Furthermore, the ELANS study did not assess recreational screen time as suggested in previous guidelines^7^. As there are no specific public health recommendations concerning 24-h Movement Guidelines for inhabitants from Latin America, the study also relied on the Canadian movement guidelines. On the other hand, there are several strengths of the present study. We used a large sample size from eight Latin American countries using a rigorous quality control program to ensure high quality data across all countries. Furthermore, we present objective measures to assess MVPA and sedentary behavior, which is rare in Latin America^31,53^.

## Conclusion

We showed that less than 2% of adults met all three movement guidelines. Effective strategies are, therefore, needed to promote healthy lifestyles in Latin American adults. Particularly, engagement in more MVPA and less sedentary behavior along with adequate sleep should be encouraged in Latin American adults, mainly women from urban areas.

To improve empirical evidence for developing evidence-based 24-h movement guidelines for the Latin American adult population, future research is recommended that uses perspective and intervention designs to examine the associations between compliance with 24-h movement guidelines and relevant health outcomes.

## Data Availability

The datasets generated and/or analyzed during the current study are not publicly available due the terms of consent/assent to which the participants agreed but are available from the corresponding author on reasonable request. Please contact the corresponding author to discuss availability of data and materials.
